# Predictive Correlation Between Hardness and Tensile Properties of Submerged Arc Welded API X70 Steel

**DOI:** 10.3390/ma18194482

**Published:** 2025-09-25

**Authors:** Ali Lahouel, Sameh Athmani, Amel Sedik, Adel Saoudi, Regis Barille, Lotfi Khezami, Ahlem Guesmi, Mamoun Fellah

**Affiliations:** 1 Surface Engineering Laboratory (L.I.S), Faculty of Sciences, Badji Mokhtar-Annaba University, P.O. Box 12, Annaba 23000, Algeria; ali.lahouel@univ-annaba.dz; 2 Centre de Recherche Scientifique et Technique en Analyses Physico-Chimiques CRAPC, Zone Industrielle Bou-Ismail, BP 384, Tipaza 42001, Algérie; samehathmani@yahoo.fr (S.A.); amelsedik2015@gmail.com (A.S.); saoudi_dl@yahoo.com (A.S.); 3 MOLTECH-Anjou, Université d′Angers/UMR CNRS 6200, 2 Bd Lavoisier, 49045 Angers, France; 4 Department of Chemistry, College of Science, Imam Mohammad Ibn Saud Islamic University (IMSIU), P.O. Box 5701, Riyadh 11432, Saudi Arabia; amalkasme@imamu.edu.sa; 5 Mechanical Engineering Department, Abbes Laghrour-Khenchela University, P.O. Box 1252, Khenchela 40004, Algeria; mamoune.fellah@univ-khenchela.dz

**Keywords:** statistical modeling, correlation, Vickers hardness, tensile test, submerged arc welding, API X70

## Abstract

This research investigates the statistical correlation between Vickers hardness and tensile properties of helical submerged arc welded high-strength low-alloy (HSLA) API X70 pipeline steel. Tensile tests were performed on cross-weld joints from 138 pipe specimens. Vickers hardness measurements were also conducted on 138 samples to evaluate the hardness distribution across the base metal, fusion zone, and heat-affected zone. Results show that the fusion zone exhibits the highest hardness, correlating with enhanced tensile strength (R^2^ = 82%). Linear regression models indicate that base metal hardness significantly influences yield strength (R^2^ = 71%), while moderate negative correlations exist with elongation (R^2^ = 54%). These findings suggest that hardness measurements can serve as a non-destructive predictive tool for tensile properties, improving weld quality and mechanical performance. This research provides empirical models that enhance the application of API X70 in critical engineering applications, improving pipeline safety and reliability.

## 1. Introduction

The properties of tensile and hardness are essential concepts in materials science and engineering that play a crucial role in material design, manufacturing, and performance evaluation [1]. A material’s tensile strength refers to its ability to resist deformation under tension, while hardness measures its resistance to indentation, scratching, or deformation [2,3].

Understanding the relationship between mechanical properties such as tensile strength and hardness is crucial [4,5]. These properties significantly influence the selection, processing, and application of materials in various industries, including construction, automotive, and aerospace [6,7]. Specifically, optimizing these properties for welded pipeline steels like API X70, which are extensively used in the oil and gas industry, is essential for ensuring durability and performance under extreme conditions [8,9].

Several studies have investigated the relationship between tensile strength and hardness in various materials [5,10]. Costa et al. explored stress–hardness relationships in AA 6082-T6 aluminum alloy welds, showing that linear correlations between local hardness and tensile properties can be used to assess plastic properties in welds, aiding in process optimization [11]. Li et al. showed that hardness measurements could effectively predict mechanical properties in aluminum alloy 2219 and its welded joints, benefiting the aerospace industry [12]. Similarly, Rajakumar and Balasubramanian demonstrated that hardness is inversely proportional to grain size and directly proportional to tensile strength in AA1100 aluminum alloy welds [13]. Tiryakioglu et al. also developed empirical equations for aluminum alloy 7010, offering a non-destructive testing method for aerospace applications [14,15].

Another study by Wei et al. found that microhardness in electron beam welded TC4-DT joints reliably predicts tensile properties, providing valuable insights for the aerospace industry [16]. Borisenko et al. established that both ultimate strength and yield point in niobium alloys correlate linearly with hardness, facilitating efficient assessments of high-temperature materials [17]. The research by Jayme and Palmer demonstrated that microhardness can predict tensile strength in additively manufactured Ti-6Al-4V components, improving inspection protocols [18]. Moreover, Krishna et al. provided practical methods for estimating tensile properties in copper alloys through established linear correlations between hardness and strength [19].

Several publications have appeared in recent years documenting the relationship between hardness and tensile in welded joints of high-strength low-alloy steels. One of the first studies, presented by Hashemi, investigated the statistical correlation between strength and hardness in API X65 steel using Vickers hardness data from 100 tested pipes [20]. The research developed an empirical equation and demonstrated that hardness measurements could predict yield strength with reasonable accuracy. This contribution enhances the understanding of how hardness can serve as a non-destructive predictor of yield strength in pipeline steels, facilitating efficient quality control in industrial applications [21].

Pamnani et al. studied the mechanical properties of micro-alloyed HSLA steel weld joints using automated ball indentation (ABI) [22]. They evaluated the variations in yield strength, tensile strength, and strain hardening components across different welding techniques, including SMAW, SAW, FCAW, and A-GTAW [23,24]. The research demonstrated that ABI provides reliable estimates of tensile properties that are consistent with conventional tensile tests [22]. This approach enhances the understanding of mechanical property variations across weld joints, offering valuable insights into optimizing welding techniques for improved strength and ductility in high strength low alloy steels [22].

Castelluccio et al. introduced an SEM-based method combined with image correlation techniques for evaluating tensile properties in the HAZ of welded API X65 steel, offering a reliable tool for structural integrity assessments [25]. This method allows for precise measurement of local stress–strain properties at a sub-millimeter scale, providing detailed insights into the mechanical behavior of the coarse-grain HAZ (CGHAZ) under different heat inputs [9]. The study demonstrated that the proposed technique could accurately capture the tensile behavior of HAZ regions, overcoming limitations of traditional hardness measurements and notched bar testing [25].

Midawi et al. investigated yield strength mismatch in API X80 pipeline steel welds using an instrumented indentation technique [26]. The research demonstrated that this technique could accurately estimate yield strength in welds and heat-affected zones, showing results within 4.6% agreement with conventional tensile testing [26]. This method allows for precise local measurements of yield strength in narrow zones like the heat-affected area, providing a practical tool for assessing strength mismatch in strain-based pipeline designs where conventional testing methods are limited [27].

Using micro-hardness tests, Peng et al. characterized the heterogeneous constitutive relationship of submerged arc welded joints in Q345 HSLA steel [28]. They developed a new method to predict local constitutive properties across the welded zone by determining the strain hardening exponent from micro-hardness values [29]. The study showed that the predicted properties align closely with experimental results from micro-tensile tests, providing a practical approach for assessing the mechanical behavior of welded joints [26].

In recent years, machine learning (ML) approaches (including neural networks, fuzzy logic systems, etc.) have been increasingly used to predict mechanical properties of welded joints with high accuracy [30,31,32,33,34,35,36]. However, these methods are often complex, require significant tuning, and are typically considered “black-box” models, offering limited interpretability. In contrast, this study adopts a transparent and statistically grounded linear regression approach. By leveraging a large dataset collected across various weld zones in API X70 joints, our models provide interpretable and practically useful correlations between hardness and tensile properties.

While prior studies, such as those by Hashemi on API X65 [20] and Saoudi et al. on API X70 [37], have investigated the relationship between hardness and tensile strength, their analyses were limited to the base metal. In contrast, the present study uniquely focuses on the correlation between hardness and tensile properties (YS, TS, EL, and YTR) of the submerged arc welded joints of API X70. To our knowledge, no previous research has systematically collected tensile data from welded joints of API X70 at this scale. By analyzing 138 samples, this work will provide a more comprehensive understanding of how mechanical properties correlate in real welded structures, offering more practical insights for quality control and pipeline design.

This research aims to bridge this gap by investigating the interplay between tensile strength and hardness in welded X70 steel. Such an investigation is critical for streamlining weld quality assessments, as hardness is often used as an estimator for tensile strength in engineering applications [38]. By focusing on welded X70 joints, this study will provide insights into how welding parameters and microstructural changes influence mechanical properties, complementing existing studies that primarily examine base metals or isolated weld zones [39].

The findings of this research will contribute to optimizing the use of API X70 steel in critical applications such as pipeline construction, where both tensile strength and hardness are paramount. Understanding these correlations can enhance material selection processes, improve quality control measures, and ultimately ensure safer and more efficient pipeline systems [20].

## 2. Materials and Methods

### 2.1. Materials

This study investigates cross-weld joints of high-strength low-alloy steel API X70 used in gas transportation pipelines. The API X70 pipes have an outside diameter of 711 mm and a wall thickness of 14.3 mm. The pipes were formed using the spiral welding technique, with original coils produced through the thermo-mechanical controlled rolling (TMCR) process [40].

The chemical composition of the API X70 steel was determined using optical emission spectroscopy Spectrolab LAVM 11A (SPECTRO, Kleve, Germany). Sample preparation and test procedures were performed according to API 5L recommendations [41]. The average chemical composition of API X70 is presented in Table 1.

The sum of Nb, V, and Ti contents in this study was 0.074%, which is well within the maximum limit of 0.15% specified by the API 5L standard [41].

### 2.2. Welding Procedure

For the welding of large-diameter, high-pressure API X70 pipeline steels, a narrow-gap, double-sided, single-pass submerged arc welding technique was selected. The welded joints featured a V-shaped bevel, as detailed in Table 2, which presents the relevant welding parameters. Submerged arc welding, known for its capability to deliver high deposition rates, offers the advantages of both increased welding speed and enhanced productivity; in this study, a travel speed of approximately 0.72 m/min was maintained.

The procedure utilized an S2Mo copper-coated filler wire with a 4mm diameter. Shielding was achieved using Eliflux BFB (GEKA, Istanbul, Turkey), an agglomerated flux with a grain size ranging from 4 mm to 15 mm. During welding, the electrical arc is established between the pipeline and the wire, remaining hidden below the flux layer. The flux fulfilled multiple vital roles: it protected the molten weld pool from atmospheric oxidation, facilitated the transfer of chemical elements between the weld metal and slag, and acted as both a deoxidizer and a cleaner for the molten metal due to its basic characteristics. The welding parameters were optimized based on preliminary trial welds to ensure consistent weld quality and repeatability.

### 2.3. Mechanical Characterization

#### 2.3.1. Tensile Tests

**Figure 1:**
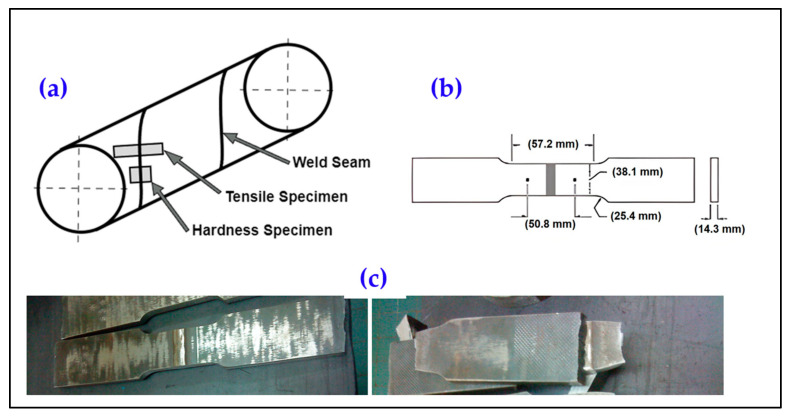
Schematic representation of (**a**) tensile and hardness sampling from welded pipes, (**b**) dimensions of standard tensile samples, and (**c**) tensile specimens before and after tensile fracture.

Tensile tests were conducted to evaluate the mechanical properties of the API X70 steel. Full-thickness flat strip specimens, each with a thickness of 14.3 mm, were used. Specimens were cut from the welded pipes following the specified position and direction, as shown in Figure 1. The specimens were prepared by flame cutting and flattening using a hydraulic press to remove initial curvature. While this process may slightly redistribute or relieve some residual stresses, it was performed carefully to avoid inducing additional plastic deformation. The main purpose was to standardize specimen geometry for accurate tensile testing.

These flat, dog bone-shaped samples were used to measure yield strength (YS), tensile strength (TS), and elongation (EL) according to the EN 10002-1 standard [42].

The tensile experiments were performed at room temperature using a 1200 kN Zwick (Zwick Roell, Ulm, Germany) tensile testing machine with a gauge length of 50.8 mm. A total of 138 tensile tests were conducted, and the mechanical properties of the cross-weld specimens were evaluated against the industry requirements outlined in the API 5L standard [41].

#### 2.3.2. Hardness Experiments

**Figure 2:**
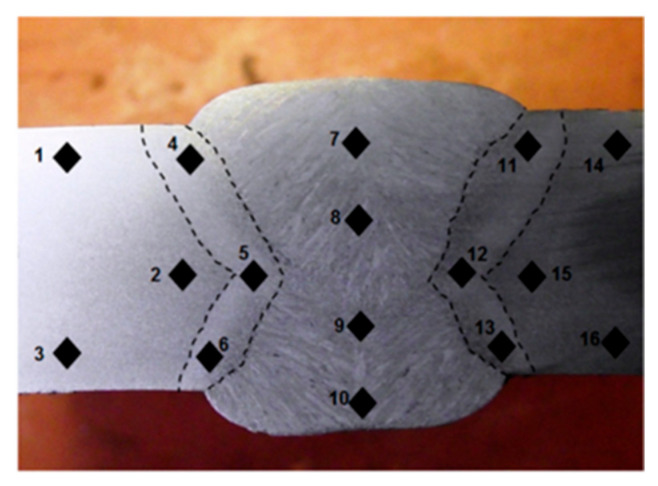
Vickers hardness measurements in various subzones of weld joints. BM hardness (average of data points 1, 2, 3, 14, 15, 16), HAZ hardness (average of 4, 5, 6, 11, 12, 13) and FZ hardness (average of 7, 8, 9, 10).

Hardness tests are essential for ensuring the structural integrity of welded steel pipes [28]. The Vickers hardness test was used to measure the hardness levels of the API X70 steel in different regions of the weldments [20]. Hardness measurements were carried out on the base metal, heat-affected zone (HAZ), and fusion zone (FZ) using a Zwick Vickers hardness tester (Zwick Roell, Ulm, Germany) with a 10 kgf load (HV10). The Vickers hardness test with a 10 kgf load (HV10) was selected in accordance with standard practice for welded joint characterization, as recommended by both the API 5L specification and several peer-reviewed studies on pipeline steels [20,28]. HV10 provides a practical balance between spatial resolution and depth of penetration which makes it suitable for distinguishing hardness variations across different weld subzones (base metal, HAZ, and fusion zone), while minimizing the influence of microstructural heterogeneity over too wide an area. In total, 138 weld joints were tested for the hardness measurement. Specimens were cut from the pipe body (Figure 2) and polished using a series of emery paper and diamond paste, followed by etching with 2% Nital solution for macrographic examination, as shown in Figure 2, prior to the hardness tests. Hardness measurements were conducted at various subzones of the weld joints, with specific attention to potential hard spots that could initiate cracks. According to the API 5L standard, any hard spot with a hardness level greater than or equal to 250 HV should be rejected.

#### 2.3.3. Microstructural Analysis

**Figure 3:**
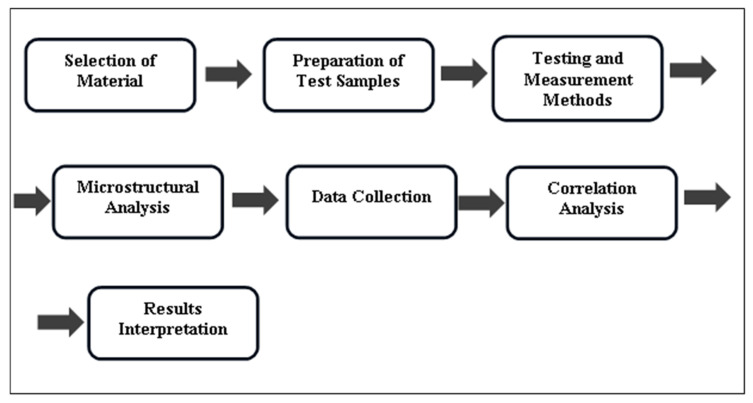
Flow chart of the study.

Using optical microscopy, microstructural changes in the weld subzones, including the base metal (BM), heat-affected zone (HAZ), and fusion zone (FZ), were examined. Samples were prepared through standard metallographic polishing and etched in a 2% Nital solution. The obtained samples were examined using a Nikon Eclipse LV100ND optical microscope (Nikon Corporation, Tokyo, Japan). Figure 3 illustrates the flow chart of our study.

## 3. Results

### 3.1. Tensile Test Results

The descriptive statistics, grouped in Table 3, for each tensile characteristic of weld metal provide valuable insights into its mechanical properties. It summarizes all test results from tensile experiments. Statistical analysis has been conducted on the yield strength (YS), tensile strength (TS), elongation (EL), and yield-to-tensile ratio (YTR). In Table 2, N refers to the number of tests conducted for each property. Mean represents the average value of the measured property. SD stands for standard deviation, indicating the variability of the measurements. Minimum and Maximum denote the lowest and highest recorded values, respectively. The API 5L column shows the relevant standard limits for tensile strength as defined by the API 5L specification; “NA” indicates that no specified limit exists in the standard for that property.

From Table 3, the average yield strength recorded is 620.76 MPa, with a standard deviation of 22.72 MPa. The minimum and maximum yield strength values (570.90 and 678.10 MPa) show some variability. However, overall, the values are relatively clustered close to the mean, which is a good sign for the consistency of mechanical properties [43]. Regarding tensile strength (TS), which is the primary focus of API 5L specifications, the weld metal demonstrates a mean value of 689.954 MPa with a standard deviation of 12.1865 MPa. The observed minimum and maximum values of 652.7 MPa and 723.8 MPa fall well within the API 5L specified range of 570–760 MPa. This compliance is crucial for ensuring the material meets industry standards and requirements [41].

The mean percentage elongation is 18.57%, showing the material’s ductility. However, the data have a significant spread with a standard deviation of 6.368%. The wide range from a minimum of 2.50% to a maximum of 28.90% indicates that some specimens demonstrated far more ductility than others. This spread in elongation values could be attributed to variations in the welding process or localized differences in material properties [44]. The yield-to-tensile ratio of weld metal directly reflects its ability to undergo significant plastic deformation and indicates its susceptibility to fracture. The mean yield-to-tensile ratio is about 89.97%, with a standard deviation of 2.671%. With YTR values ranging from a low of 83.064% to a high of 96.227%, most weld metal specimens possess a high proportion of their ultimate tensile strength as yield strength, which implies that the material is less ductile and hard [44].

While API 5L specifically sets requirements for tensile strength, the other mechanical properties presented in the table are equally crucial for a comprehensive understanding of the weld metal’s behavior [41]. The data suggests that the weld metal in API X70 steel demonstrates consistent strength properties that meet the required specifications, with some variability in ductility as indicated by the elongation values. This information is valuable for assessing the overall quality and performance of the welded joints in API X70 steel applications.

The provided data on API X70 weld metal’s mechanical properties align with recent research while highlighting areas requiring deeper consideration. The tensile strength (689.95 ± 12.19 MPa) demonstrates better consistency than yield strength (620.76 ± 22.72 MPa), aligning with findings that proper Welding Procedure Specifications (WPS) maintain stable tensile properties [45]. However, the elongation range (2.5–28.9%) shows significant variability, which is consistent with observed variations in submerged arc welding (SAW) applications. This variability can be attributed to factors like heat input effects and microstructural heterogeneity [46]. The high yield-to-tensile ratio (89.97 ± 2.67%) indicates that the material is less ductile and harder, which aligns with modern pipeline steel designs emphasizing strength over ductility. However, recent studies suggest this metric might mask critical brittleness in heat-affected zones [6]. To address these issues, recent advances highlight the importance of process control, including parameter optimization and microstructure engineering to improve ductility. Additionally, there is a growing need for supplementary testing protocols to address localized property variations overlooked by current standards [25].

### 3.2. Hardness Test Results

Table 4 summarizes the average data for 138 hardness tests in each of the three sub-zones (BM, FZ, HAZ) of API X70 weld joints.

#### 3.2.1. Fusion Zone Hardness and Microstructure

From Table 4, the fusion zone exhibits the highest average hardness (227 HV) among the three zones. This is expected as the FZ undergoes rapid heating, fusion, cooling, and solidification during welding, leading to microstructural changes that increase hardness. It also shows the most significant variation in hardness with the highest standard deviation (3.28), indicating potentially uneven cooling or compositional variations within the FZ. Notably, the maximum hardness value of 234 HV in the FZ is well below the 250 HV limit set by API 5L, suggesting that the fusion zone meets the hardness requirement. The range of hardness values from minimum 209.25 HV to maximum 234.00 HV indicates all samples fall within a tight range, which can indicate uniform material properties in the fused zone.

The observation that the FZ exhibits the highest average hardness (227 HV) is consistent with recent studies, which attribute this to rapid heating, cooling, and solidification during welding. Research has shown that the FZ often contains refined microstructures such as proeutectoid ferrite, acicular ferrite, and fine pearlite (Figure 3), which contribute to its higher hardness values. However, new findings reveal that heat treatments can significantly alter these properties. For instance, prolonged heat treatments at 550 °C led to grain growth and carbide precipitation, which reduced hardness over time. Hardness in the FZ decreased from 230 HV at 1 h to 170 HV at 5 h due to these microstructural changes [47]. Additionally, the variation in hardness within the FZ, as indicated by its standard deviation (3.28), may also be influenced by compositional segregation or uneven cooling rates. Studies have shown that slower cooling rates promote more uniform microstructures, whereas rapid cooling can introduce localized variations in hardness due to differential phase transformations [48].

#### 3.2.2. Base Metal and Heat-Affected Zone Characteristics

**Figure 4:**
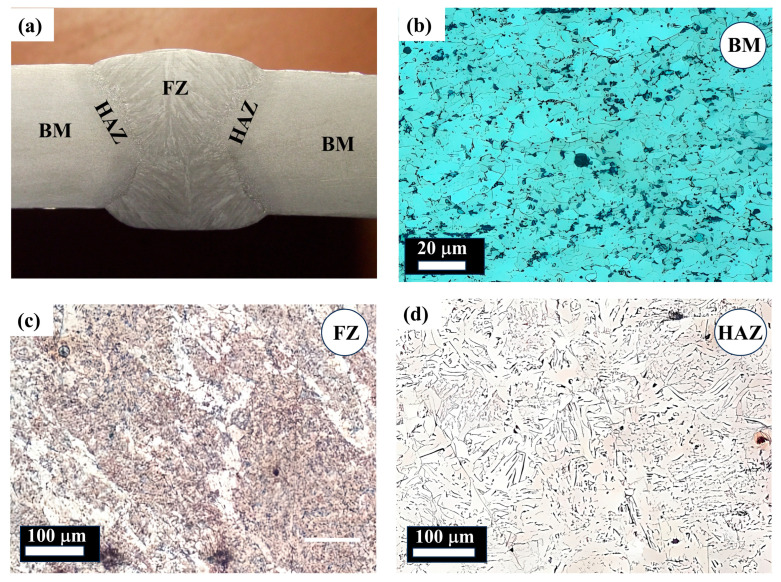
Microstructure of the test material (**a**) macrograph of the welded joint, (**b**) microstructure of the base metal, (**c**) microstructure of the fusion zone, and (**d**) microstructure of the heat-affected zone.

The base metal (222 HV) and the heat-affected zone (222 HV) have similar average hardness values (Table 4). The similar average hardness values of the BM (222 HV) and HAZ (222 HV) suggest that the welding process has a less pronounced effect on these zones compared to the FZ (Table 4). This observation is supported by studies showing that the BM’s equiaxed ferritic–pearlitic matrix (Figure 4), provides a stable baseline for mechanical properties [47,49]. However, it is worth noting that compositional differences between the BM and the HAZ can influence their response to welding and subsequent heat treatments.

The HAZ, however, exhibits a broader range of hardness values (210 to 232 HV) than the BM (216 to 229 HV) despite having a slightly lower standard deviation (Table 4). This broader range in the HAZ could be attributed to the varying thermal cycles experienced at different distances from the fusion line. Similarly to the FZ, both the BM and HAZ have maximum hardness values well within the acceptable limit of API 5L [48,50]. Figure 4 shows a mixture of acicular ferrite, polygonal ferrite, and bainite microstructures. The data indicates that the welding process significantly influences the hardness of the API X70 steel, with the fusion zone experiencing the most significant changes. The variation in hardness within each zone highlights the importance of understanding and controlling the welding parameters to achieve desired mechanical properties in the weld joints [37]. Crucially, all three subzones of the weld joints appear to comply with the API 5L hardness requirement, as none of the maximum values exceed the 250 HV limit.

### 3.3. Relationship Between Tensile and Hardness

Measuring the tensile strength of pipe materials is a complex and time-consuming process that involves multiple steps [20]. Initially, a test plate is flame-cut to create the required specimens, which are then flattened, machined, and carefully prepared for testing. In contrast, conducting a hardness test is significantly quicker and easier [51]. Therefore, utilizing hardness measurements to predict material strength can offer a more efficient method for assessing the mechanical quality of weldment specimens [52].

The relationship between tensile properties and hardness is vital for predicting material performance in welded joints. Hardness tests, being simpler and faster than tensile tests, present an attractive alternative for estimating tensile strength [12]. This study aims to establish a correlation between hardness and tensile properties to streamline the assessment of weld quality.

**Figure 5:**
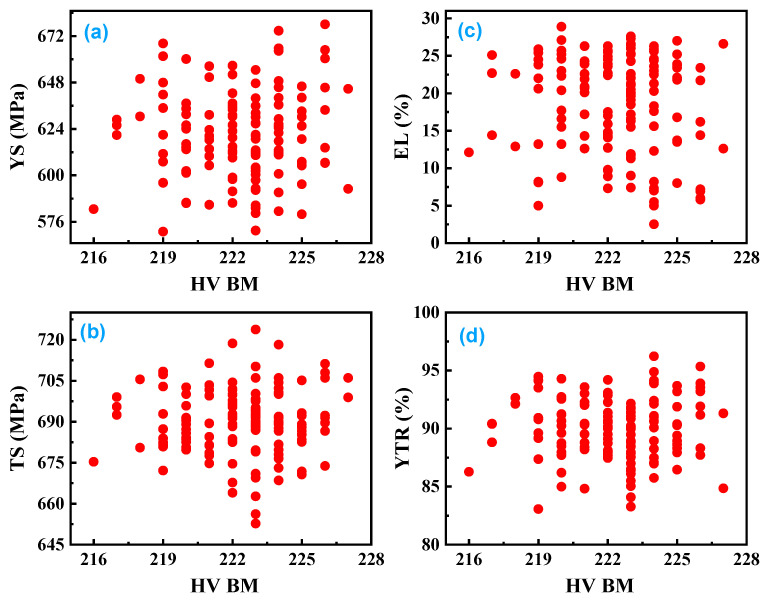
Scatter plot of tensile properties of weld metal: (**a**) YS, (**b**) TS, (**c**) EL, and (**d**) YTR, versus hardness of the base metal in API X70 steel.

Figure 5 displays scatter plots showing how the yield strength (YS), tensile strength (TS), elongation (EL), and yield-to-tensile ratio (YTR) of the weld metal relate to the hardness of the base metal (BM) in API X70 steel. The data indicate that even at similar hardness levels in the base metal, there is significant scatter in the tensile properties of the weld metal. For example, YS and TS values fluctuate noticeably for a given hardness value, and elongation and YTR also show wide variation. The observed scatter likely points to underlying factors such as microstructural heterogeneity and residual stresses that are not captured by hardness measurements alone.

**Figure 6:**
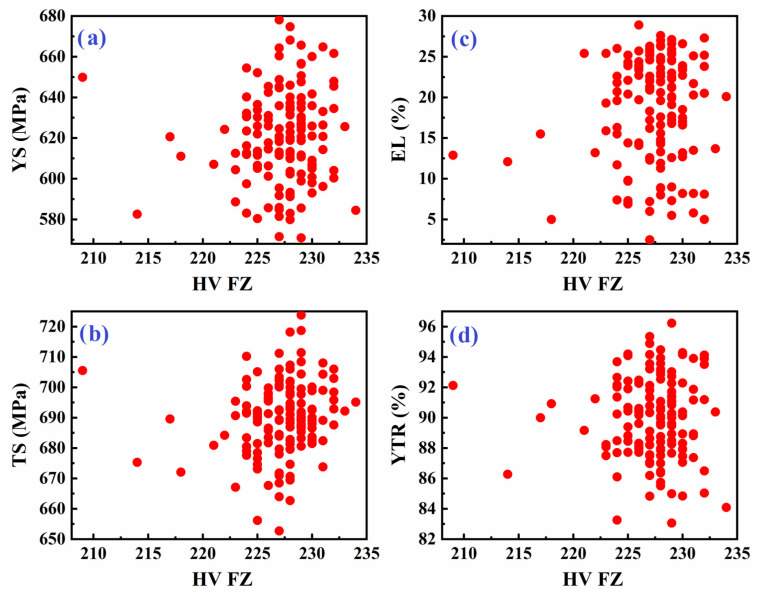
Scatter plot of tensile properties of weld metal: (**a**) YS, (**b**) TS, (**c**) EL, and (**d**) YTR, versus hardness of the fusion zone in API X70 steel.

Figure 6 presents similar scatter plots but correlates the tensile properties of the weld metal with the hardness of the fusion zone (FZ). As with the base metal, the data show considerable variability in YS, TS, EL, and YTR for a given fusion zone hardness. While there may be general trends (e.g., higher hardness sometimes correlates with higher strength), the relationship is inconsistent. This inconsistency further supports the idea that factors beyond simple hardness—such as local variations in microstructure, phase distribution, and residual stress from welding—play a significant role in determining the final tensile properties of the weld metal.

**Figure 7:**
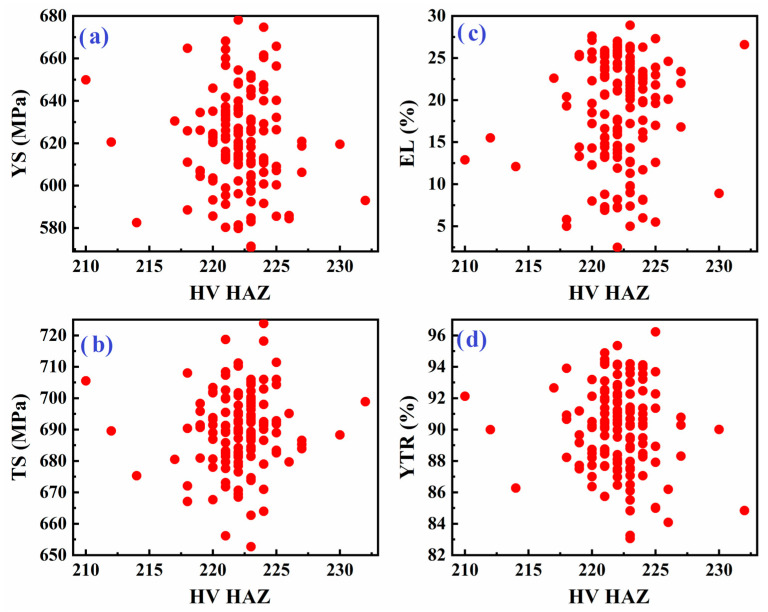
Scatter plot of tensile properties of weld metal: (**a**) YS, (**b**) TS, (**c**) EL, and (**d**) YTR, versus hardness of the heat-affected zone in API X70 steel.

Figure 7 explores the relationship between weld metal tensile properties and the hardness of the heat-affected zone (HAZ). The scatter plots again reveal a substantial data spread; for any given HAZ hardness, the tensile properties of the weld metal can differ widely. The variability may be attributed to the complex thermal cycles experienced in the HAZ, leading to microstructural changes and residual stresses that impact mechanical performance independently of measured hardness.

Overall, the observed inconsistencies could be attributed to several factors, including heterogeneity in the microstructure among the various weldment sub-zones and residual stresses introduced during welding. Depending on welding parameters, these stresses can affect tensile properties even if the hardness remains similar. Additionally, hardness values were computed as the algebraic average of six data points in the base metal, four data points in the fusion zone, and six data points in the heat-affected zone.

**Figure 8:**
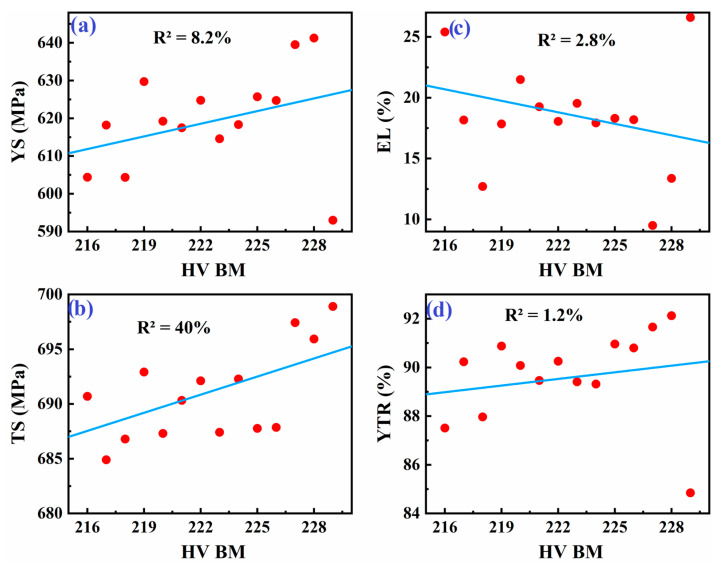
Linear fit between tensile properties of weld metal: (**a**) YS, (**b**) TS, (**c**) EL, and (**d**) YTR, and hardness of base metal in welded API X70 steel.

**Figure 9:**
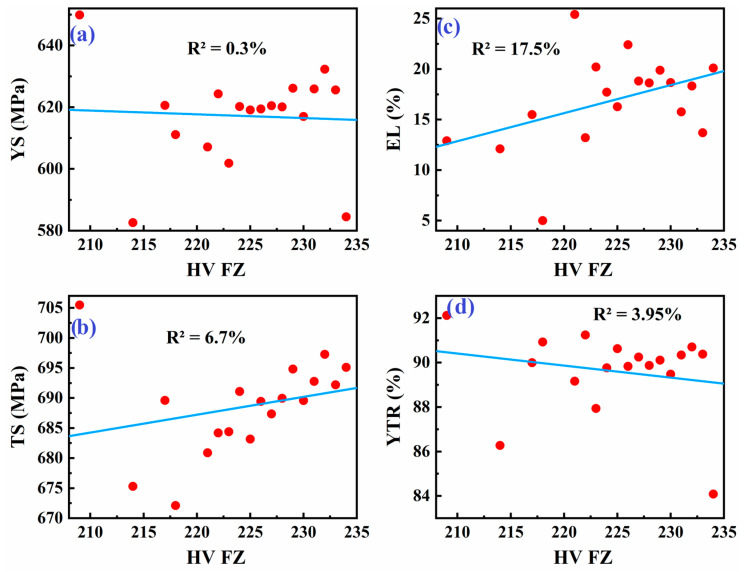
Linear fit between tensile properties of weld metal: (**a**) YS, (**b**) TS, (**c**) EL, and (**d**) YTR, and hardness of fusion zone data in welded API X70 steel.

**Figure 10:**
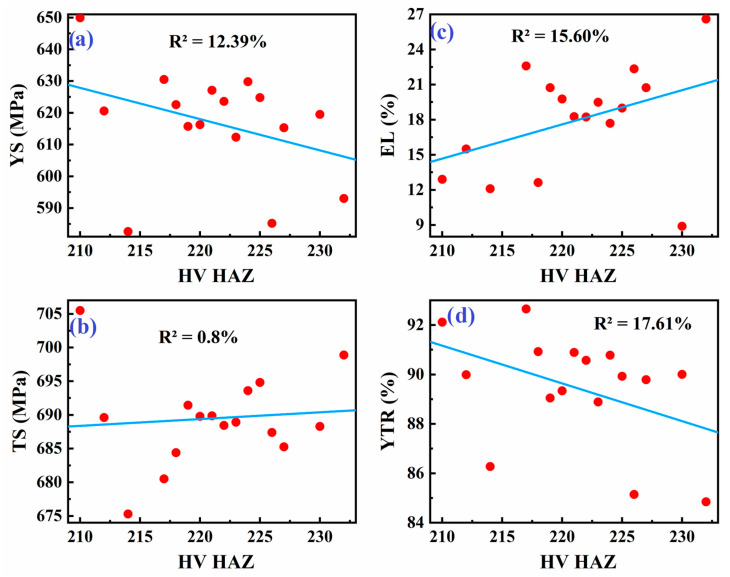
Linear fit between tensile properties of weld metal: (**a**) YS, (**b**) TS, (**c**) EL, and (**d**) YTR, and hardness of the heat-affected zone data in welded API X70 steel.

As shown in Figure 8, Figure 9 and Figure 10, averaging the data points for each hardness level helps simplify the data and identify potential trends. This approach can reveal an underlying relationship between hardness and tensile properties of the weld metal, although it may not capture the full complexity of the situation.

Figure 8 presents linear regression models correlating the weld metal’s tensile properties with the hardness of the base metal (BM) in welded API X70 steel. Initially, the coefficient of determination (R^2^) for these models ranged from 1.2% to 40%, indicating very weak predictive power due to high data variability and the presence of outliers. Outliers were identified based on standardized residuals from the regression model, with points exceeding ±2 standard deviations considered outliers.

Figure 9 illustrates the correlation between tensile properties of the weld metal and the hardness of the fusion zone (FZ). Similarly to Figure 8, this graph shows a linear relationship but with a similarly low R^2^ value, emphasizing a weak correlation. The fusion zone in welded steel often exhibits a unique microstructure due to rapid cooling and different thermal conditions during welding. This leads to variations in hardness, which in turn influences tensile properties. The relatively low correlation seen in Figure 9 is expected as the fusion zone can have varied microstructural features like martensite or bainite, which can be very sensitive to cooling rates and welding parameters. The literature supports this finding, suggesting that the correlation between hardness and tensile strength in the fusion zone is not always strong, especially in high-strength steels like API X70, where complex phases form during the welding process [20,37].

In Figure 10, the relationship between the tensile properties of weld metal and the hardness of the heat-affected zone (HAZ) is presented. Similarly to the previous figures, this graph shows a weak linear relationship with an R^2^ ranging from 1.2% to 40%.

The heat-affected zone (HAZ) is crucial in determining the mechanical properties of welded joints. It is known for its heterogeneity, with varying hardness across different regions of the HAZ due to temperature gradients during welding. The low correlation in Figure 10 is in line with the literature, which indicates that the HAZ often exhibits a complex relationship between hardness and tensile properties. Variations in grain structure, phase transformations, and the presence of residual stresses all contribute to the variability observed in tensile properties.

The relationship between hardness and tensile strength in steels is generally expected to be somewhat linear, but it can be influenced by various factors such as microstructure, heat treatment, and welding parameters. A low R^2^ value, as shown in Figure 8, Figure 9 and Figure 10, is consistent with reports in the literature where the correlation between hardness and tensile properties in welded materials can be weak due to complex interactions at the microstructural level. For instance, research on the welding of steels, including X70, has pointed out that although hardness is often correlated with yield strength and tensile strength, the effect is not straightforward due to the heterogeneity in the weld and heat-affected zones (HAZs). As noted by previous studies, outliers and variations in microstructure lead to data scattering, weakening the strength of the correlation [20,53]. In this study, the removal of outliers based on standardized residuals (>±2) was carried out to improve the reliability and interpretability of the regression models. This approach enhances model fit by minimizing the influence of extreme values that do not conform to the overall data trend [54,55].

To improve the reliability and predictive strength of the regression models, a three-step data cleaning procedure was employed based on standardized (studentized) residuals. Outliers were defined as observations with residuals exceeding ±2.

▪ Step 1 (Model 1): Initial regression models were constructed using averaged data (e.g., mean values of grouped hardness or tensile properties).
▪ Step 2 (Model 2): Outliers were identified in Model 1 using the standardized residual criterion. If present, these outliers were removed, and the model was rebuilt using the cleaned data.
▪ Step 3 (Model 3): A second round of residual analysis was conducted on Model 2. If additional outliers were detected, they were removed to generate a final cleaned model.

In cases where no outliers were detected after either Step 1 or Step 2, the cleaning process was concluded at that stage. This approach ensured that outlier removal was performed only when necessary, preventing over-filtering and preserving the statistical integrity of the dataset. The results of data cleaning are presented by Table 5.

#### 3.3.1. Hardness Correlation with YS and TS

**Figure 11:**
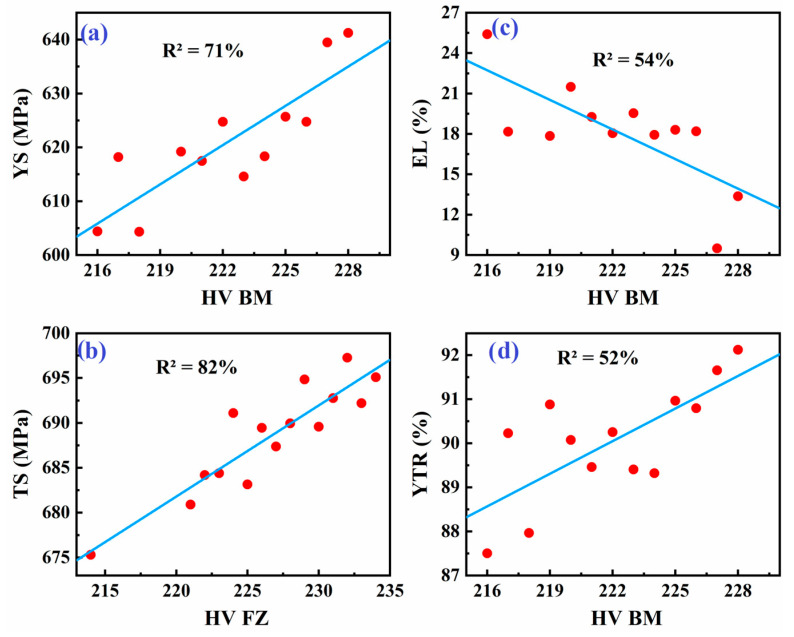
Best linear models obtained after data cleaning. (**a**) YS of weld metal versus HV BM, (**b**) TS of weld metal versus HV FZ, (**c**) EL of weld metal versus HV BM, and (**d**) YTR of weld metal versus HV BM.

After data cleaning, the regression models demonstrated significantly improved R^2^ values: 71% for yield strength (YS) and 82% for tensile strength (TS), indicating strong predictive capabilities (Figure 11 and Table 5). The regression models are given by Equations (1) and (2):

YS = 81 + 2.43 × (HV BM)(1)

TS = 458 + 1.01 × (HV FZ)(2)

This improvement mirrors findings in studies using machine learning techniques, such as fuzzy logic systems and neural networks, which emphasize the importance of preprocessing data to enhance prediction accuracy. For example, a fuzzy logic approach achieved a coefficient of determination of 99% for predicting tensile strength in TIG mild steel welds [31]. Also, neural network models predicted tensile strength with <2% error, showcasing the effectiveness of advanced prediction tools when data are refined [32].

The observed trend, where base metal hardness (HV BM) correlates strongly with yield strength (YS) (R^2^ = 71%), while fusion zone hardness (HV FZ) correlates better with tensile strength (TS) (R^2^ = 82%), can be explained by the distinct deformation mechanisms and microstructural features of each subzone. Yield strength represents the onset of plastic deformation, which is largely governed by the base metal’s microstructure, typically composed of ferrite and pearlite (Figure 4b). This structure provides a stable resistance to dislocation motion, thus linking YS closely with HV BM. In contrast, the tensile strength reflects the material’s resistance to fracture under maximum load, which is more sensitive to hard phases and strain hardening behavior present in the fusion zone. The FZ (Figure 4c) contains refined acicular ferrite and bainitic structures formed by rapid solidification, which enhance hardness and ultimate strength. These microstructural differences justify the stronger correlation between HV FZ and TS observed in our models. This interpretation aligns with previously reported findings on welded high-strength steels [39,53,56].

It is important to note that the predictive models developed in this study are empirical and specifically tailored to submerged arc welded joints in API X70 steel under controlled welding parameters. While strong correlations were observed between hardness and both yield strength (R^2^ = 71%) and tensile strength (R^2^ = 82%) after data cleaning, these relationships are inherently dependent on the microstructural characteristics of the material and welding process [32,35]. As such, the applicability of these models to other steels or welding conditions, particularly those with different alloying elements, cooling rates, or grain structures, may be limited. Furthermore, defects such as pores or cracks, which can significantly reduce tensile performance, are not captured by hardness measurements alone. Therefore, while the models offer a useful and efficient non-destructive estimation tool for preliminary assessments, they should not replace full mechanical testing or defect evaluation in safety-critical applications.

#### 3.3.2. Hardness Correlation with Elongation and Yield-to-Tensile Ratio

Conversely, a moderate negative correlation between base metal hardness and weld metal elongation (R^2^ = 54%) (Figure 11), was found, described by Equation (3):

EL = 181 − 0.73 × (HV BM)(3)

Recent research supports this observation, noting that increased hardness correlates with reduced elongation due to limited plastic deformation capacity [30,32].

Additionally, the relationship between the weld joint yield-to-tensile ratio (YTR) and the base metal hardness (HV BM) shows a moderate positive correlation (R^2^ = 52%) (Figure 11). Increased hardness typically means a higher yield point, leading to a higher yield-to-tensile ratio. The linear fit is given by Equation (4):

YTR = 35 + 0.24 × (HV BM)(4)

The moderate positive correlation between yield-to-tensile ratio (YTR) and base metal hardness(R^2^ = 52%) is consistent with findings that higher hardness increases yield points, thus enhancing YTR. This ratio is a critical indicator of structural safety margins against plastic collapse. Similar trends have been observed in studies using machine learning models to predict mechanical properties based on process parameters [36].

While linear regression models provide valuable insights into the relationships between hardness and tensile properties, recent research suggests that machine learning approaches can further improve prediction accuracy. For example: active learning frameworks using Gaussian process regression have been employed to predict multiple tensile properties simultaneously with high accuracy [30]. Machine learning methods incorporating process parameters have achieved prediction accuracies up to 89%, outperforming traditional regression models [36].

Although machine learning and non-linear models may provide higher precision, they often lack interpretability and transparency. In contrast, linear regression offers a direct and physically meaningful relationship between hardness and tensile properties, making it more suitable for practical applications and preliminary assessment. Furthermore, after data cleaning, the linear models demonstrated strong correlation values (R^2^ = 71% for YS and R^2^ = 82% for TS), justifying their use in this context. Future studies will explore non-linear and data-driven models to enhance predictive accuracy where needed.

### 3.4. Relationship Between Yield and Tensile Strength of Welded Joints

**Figure 12:**
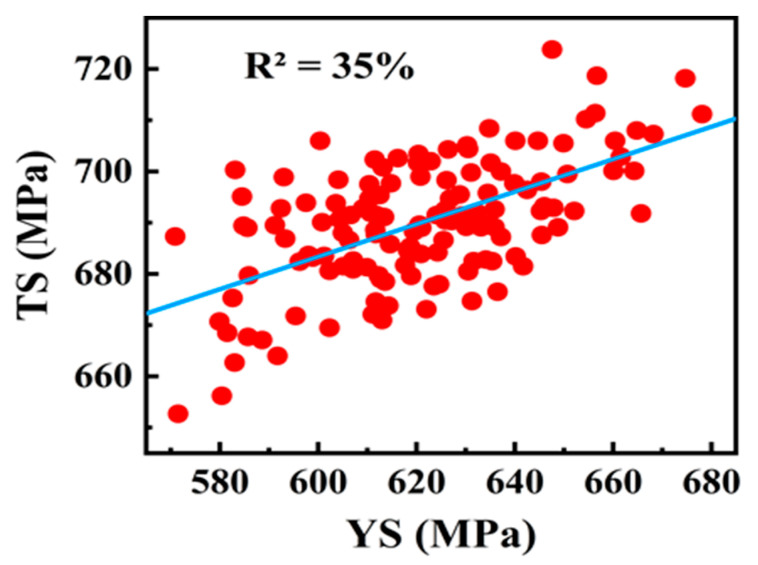
Linear correlation between yield and tensile strength of weld metal in API X70 steel.

Figure 12 illustrates the relationship between yield strength (YS) and tensile strength (TS) of weld joints in X70 steel. The data shows a general upward trend, indicating a positive but moderate correlation between the two properties. The R^2^ value of 35% suggests that approximately 35% of the variability in tensile strength can be explained by yield strength. The regression model derived from this relationship is represented by Equation (5):

TS = 493 + 0.31 × (YS)(5)

Despite the positive trend, the data exhibits substantial scatter, indicating that the relationship between YS and TS is not particularly strong. This implies that knowing the YS of the weld joint does not allow for a highly accurate prediction of tensile strength. Welding parameters, post-weld heat treatment (PWHT), and factors contributing to data scatter significantly influence tensile properties in shielded metal arc welding (SMAW) [57]. Studies indicate that moderate welding currents result in superior ultimate tensile strength (UTS) and balanced hardness, while excessive heat input reduces tensile strength due to grain coarsening, highlighting the independent influence of heat input on tensile strength (TS) versus yield strength (YS). Additionally, variability in the YS-TS relationship arises from factors like weld metal composition, joint design, and cooling rates [58,59].

The supporting literature provides further context for these findings. The study by Hashemi demonstrated a linear relationship between yield strength (YS) and tensile strength (TS), both of base metal, in API X65 steel, exhibiting relatively low scatter and a high coefficient of determination (R^2^ = 80%) [20]. This supports the notion of a positive correlation between YS and TS. Similarly, research by Saoudi et al. confirms a linear relationship between yield and tensile strength, both of the base metal in API X70 steel [37]. Their regression model, however, performed moderately with an R^2^ value of 63%. This variability suggests that the strength relationship depends on the material’s specific conditions.

Despite the moderate R^2^ value (35%), the regression model may still offer practical utility as a conservative estimator of TS from YS. This is particularly relevant in preliminary design or inspection scenarios where full mechanical testing is not feasible. While the model should not replace tensile testing where precision is required, it may support nondestructive assessments when used with appropriate safety margins.

The relationship between YS and TS in API X70 weld joints is influenced by multiple factors beyond their linear correlation. Welding parameters, material composition, joint design, and post-weld treatments all play significant roles in determining mechanical properties such as UTS and hardness. While a general positive trend exists between YS and TS, substantial scatter underscores the complexity of these interactions. Future research should focus on isolating these variables to improve predictive models for weld metal performance.

## 4. Discussion

This study investigated the statistical relationship between Vickers hardness and tensile properties in submerged arc welded (SAW) API X70 pipeline steel. Although the general correlations between tensile strength, yield strength, and hardness are well established, our findings offer new contributions to the field, particularly concerning the welded regions of API X70 steel which is a topic that remains underrepresented in the current literature. The dataset used in this work, comprising 138 welded joints, represents one of the most extensive in similar studies and enables more statistically reliable conclusions.

Unlike many prior works that either focus on the base metal or use a limited number of samples, this study emphasizes the mechanical behavior of cross-welded regions. We implemented a data cleaning process, including outlier removal, which substantially improved the strength of our regression models. After this preprocessing, the correlation coefficients increased significantly, reaching R^2^ values of 71% for yield strength and 82% for tensile strength. This highlights the importance of data refinement in establishing reliable predictive models. Furthermore, the study provides insight into the distinct mechanical responses of different weld subzones. Specifically, we observed that base metal hardness shows a strong correlation with yield strength, while fusion zone hardness correlates more closely with tensile strength. These findings emphasize the unique contributions of each zone to the overall mechanical behavior of the weldment.

In addition to its scientific contributions, the study has practical implications for industry. The empirical models presented offer a non-destructive and time-efficient method for estimating tensile properties using hardness data alone. This is particularly valuable in large-scale pipeline production, where destructive tensile testing is often cost-prohibitive or logistically impractical. By enabling quick assessment of mechanical performance, these models can serve as effective tools for process control and quality assurance.

Moreover, the correlations developed in this study can assist in identifying regions within a weld that may be susceptible to early failure. For example, areas exhibiting higher hardness but lower elongation could indicate reduced ductility and increased brittleness, which are risk factors for crack initiation under service loading. Thus, the proposed approach not only enhances material characterization but also contributes to the early detection of potentially weak or overhardened zones. This has direct implications for ensuring structural integrity, particularly in strain-based design applications where local toughness is critical.

In summary, while the observed trends align with established mechanical behavior in steels, this study extends the understanding of how hardness can be reliably used as a predictive tool in welded API X70 pipeline steel. The integration of statistical modeling with a large experimental dataset and zone-specific analysis presents a novel and practically applicable approach for weld quality evaluation.

## 5. Conclusions

This study has established a comprehensive understanding of the relationship between Vickers hardness and tensile properties in welded API X70 steel, with significant implications for quality assessments and the optimization of welding processes in pipeline applications. By analyzing the mechanical behavior of different weldment subzones, we have identified several critical correlations that can aid in the non-destructive evaluation of weld quality. The key findings of this research are summarized as follows:

- The mean yield strength (620 MPa) and tensile strength (690 MPa) of API X70 weld joints demonstrate compliance with industry standards, while elongation shows significant variability (mean: 18.57%; range: 2.5–28.9%).
- Among the weld subzones, the fusion zone exhibited the highest average hardness (227 HV), followed by the base metal (222 HV), and the heat-affected zone (222 HV). All values remained below the API 5L limit of 250 HV.
- A strong positive correlation exists between BM hardness and YS (R^2^ = 71%).
- FZ hardness strongly correlates with TS (R^2^ = 82%).
- Moderate correlations were observed for elongation (R^2^ = 54%) and yield-to-tensile ratio (R^2^ = 52%) with BM hardness.
- Regression models suggest that Vickers hardness can reliably predict tensile properties after data cleaning, offering a nondestructive alternative to traditional tensile testing.
- These findings support using hardness tests for efficient quality control in pipeline steel applications, emphasizing the importance of controlling welding parameters to achieve desired mechanical properties.
- Future research should investigate advanced machine learning methods to improve predictive accuracy further and address localized variations in weld properties.

## Figures and Tables

**Table 1:** Average chemical composition of API X70 steel.

C	Si	Mn	P	S	Cr	Ni	Mo	Al
0.061	0.275	1.731	0.011	0	0.035	0.024	0.012	0.031
**Co**	**Cu**	**Nb**	**Ti**	**V**		**Nb+Ti+V**	**CE**	
0.004	0.019	0.062	0.011	0.001		0.074	0.175	

**Table 2:** Welding procedure parameters of helical submerged arc welding process.

Pipe Size (mm)	Bevel Configuration		Weld Seam	Wire	Flux
∅ 711 × 14.3	Angle 12° V-shape		Internal	S2Mo	BFB
			External	S2Mo	BFB
**Polarity**	**Amp. (A)**	**Volt. (V)**	**Travel speed (mm/min)**		
DC (+)	750–800	31–34	720		
DC (+)	750–800	31–34	720		

**Table 3:** Descriptive statistics of tensile properties of weld metal in API X70 steel.

Variable	N	Mean	SD	Minimum	Maximum	API 5L
YS (MPa)	138	620.768	22.7224	570.9	678.1	NA
TS (MPa)	138	689.954	12.1865	652.7	723.8	570–760
EL (%)	138	18.5688	6.48637	2.5	28.9	NA
YTR (%)	138	89.9661	2.67070	83.0642	96.2272	NA

**Table 4:** Descriptive statistics of hardness measurements in the different sub-zones of API X70 weldments.

Variable	N *	Mean	SD **	Minimum	Maximum	API 5L
HV BM (HV10)	138	222.452	2.40	216	228.5	250 max
HV FZ (HV10)	138	227.159	3.27	209.25	234	250 max
HV HAZ (HV10)	138	222.057	2.58	210	231.667	250 max

N *: Number of tests; SD **: Standard deviation.

**Table 5:** Summary statistics (n, mean, SD) for both the cleaned and uncleaned datasets.

Pair	DATA	Variable	N	Mean	SD	Min	Max	R^2^
HV BM vs. YS	Average	HV BM	14	222.5	4.18	216	229	8.23
		YS	14	619.66	13.19	593	641.27	
	First Clean.	HV BM_1	13	222	3.89	216	228	56.53
		YS_1	13	621.71	11.16	604.35	641.27	
	Second Clean.	HV BM_2	12	222.25	3.95	216	228	71.24
		YS_2	12	621.04	11.39	604.35	641.27	
HV BM vs. TS	Average	HV BM	14	222.5	4.18	216	229	39.54
		TS	14	690.9	4.26	684.9	698.9	
HV BM vs. EL	Average	HV BM	14	222.5	4.18	216	229	2.79
		EL	14	18.31	4.52	9.5	26.6	
	First Clean.	HV BM_1	13	222	3.89	216	228	28.18
		EL_1	13	17.67	4	9.5	25.4	
	Second Clean.	HV BM_2	12	222.33	3.87	216	228	53.66
		EL_2	12	18.09	3.87	9.5	25.4	
HV BM vs. YTR	Average	HV BM	14	222.5	4.18	216	229	1.16
		YTR	14	89.68	1.89	84.85	92.13	
	First Clean.	HV BM_1	13	222	3.89	216	228	52.29
		YTR_1	13	90.05	1.33	87.51	92.13	
HV FZ vs. YS	Average	HV FZ	18	224.61	6.87	209	234	0.28
		YS	18	617.13	15.84	582.6	649.9	
	Second Clean.	HV FZ_1	15	225.73	4.92	217	233	34.35
		YS_1	15	619.43	7.82	601.83	632.33	
	Second Clean.	HV FZ_2	14	225.93	5.05	217	233	40.2
		YS_2	14	620.68	6.35	607.1	632.33	
HV FZ vs. TS	Average	HV FZ	18	224.61	6.87	209	234	6.7
		TS	18	688.6	7.89	672.1	705.5	
	First Clean.	HV FZ_1	17	225.53	5.83	214	234	65.1
		TS_1	17	687.6	6.88	672.1	697.27	
	Second Clean.	HV FZ_2	15	226.6	5.33	214	234	81.9
		TS_2	15	688.51	5.98	675.3	697.27	
HV FZ vs. EL	Average	HV FZ	18	224.61	6.87	209	234	17.51
		EL	18	16.92	4.56	5	25.4	
	First Clean.	HV FZ_1	15	226.33	5.72	214	234	17.93
		EL_1	15	17.42	2.92	12.1	22.41	
	Second Clean.	HV FZ_2	14	225.86	5.61	214	234	36.75
		EL_2	14	17.68	2.84	12.1	22.41	
HV FZ vs. YTR	Average	HV FZ	18	224.61	6.87	209	234	3.95
		YTR	18	89.62	1.87	84.09	92.12	
HV HAZ vs. YS	Average	HV HAZ	16	221.25	6.18	210	232	12.39
		YS	16	616.8	17.29	582.6	649.9	
	First Clean.	HV HAZ1	15	221.73	6.08	210	232	41.07
		YS_1	15	619.08	15.2	585.2	649.9	
	Second Clean.	HV HAZ2	14	221.43	6.19	210	232	45.53
		YS_2	14	621.5	12.42	593	649.9	
HV HAZ vs. TS	Average	HV HAZ	16	221.25	6.18	210	232	0.8
		TS	16	689.5	6.96	675.3	705.5	
	First Clean.	HV HAZ1	14	222.57	5.33	212	232	18.86
		TS_1	14	689.38	4.55	680.5	698.9	
HV HAZ vs. EL	Average	HV HAZ	16	221.25	6.18	210	232	15.6
		EL	16	17.97	4.58	8.9	26.6	
HV HAZ vs. YTR	Average	HV HAZ	16	221.25	6.18	210	232	17.61
		YTR	16	89.45	2.25	84.85	92.65	
	First Clean.	HV HAZ1	15	221.73	6.08	210	232	37.31
		YTR_1	15	89.66	2.16	84.85	92.65	

## Data Availability

The original contributions presented in this study are included in the article. Further inquiries can be directed to the corresponding author.
